# Prognostic Parameters and Spinal Metastases: A Research Study

**DOI:** 10.1371/journal.pone.0109579

**Published:** 2014-10-13

**Authors:** Jefferson W. Daniel, José C. E. Veiga

**Affiliations:** Division of Neurosurgery, Santa Casa de São Paulo - Faculty of Medical Sciences, São Paulo, Brazil; Queen Mary Hospital, Hong Kong

## Abstract

**Object:**

To identify pre-operative prognostic parameters for survival in patients with spinal epidural neoplastic metastasis when the primary tumour is unknown.

**Methods:**

This study was a retrospective chart review of patients who underwent surgery for spinal epidural neoplastic metastases between February 1997 and January 2011. The inclusion criteria were as follows: known post-operative survival period, a Karnofsky Performance Score equal to or greater than 30 points and a post-operative neoplastic metastasis histological type. The Kaplan-Meier method was used to estimate post-operative survival, and the Log-Rank test was used for statistical inference.

**Results:**

A total of 52 patients who underwent 52 surgical procedures were identified. The mean age at the time of spinal surgery was 53.92 years (std. deviation, 19.09). The median survival after surgery was 70 days (95% CI 49.97–90.02), and post-operative mortality occurred within 6 months in 38 (73.07%) patients. Lung cancer, prostate cancer, myeloma and lymphoma, the 4 most common primary tumour types, affected 32 (61.53%) patients. The three identified prognostic parameters were the following: pre-operative walking incapacity (American Spinal Injury Association, A and B), present in 86.53% of the patients (p-value = 0.107); special care dependency (Karnofsky Performance Score, 10–40 points), present in 90.38% of the patients (p-value = 0.322); and vertebral epidural neoplastic metastases that were in contact with the thecal sac (Weinstein-Boriani-Biagini, sector D), present in 94.23% of the patients (p-value = 0.643). When the three secondary prognostic parameters were combined, the mean post-operative survival was 45 days; when at least one was present, the survival was 82 days (p-value = 0.175).

**Conclusions:**

Walking incapacity, special care dependency and contact between the neoplastic metastases and the thecal sac can help determine the ultimate survival of this patient population and, potentially, which patients would benefit from surgery versus palliation alone. A 2- to 3-month post-operative survival period justified surgical treatment.

## Introduction

The vertebral column is the most common site of skeletal metastases and is involved in approximately 40% of the patients who die of cancer [1]–[4]. Spinal epidural metastases as the initial manifestation of cancer comprise 20% of all spinal epidural metastases [5], [6], and the cumulative probability of spinal cord compression caused by malignant disease in the 5 years preceding death from cancer is 2.5% [6], [7]. Surgical treatment is required in 5–10% [8] of the patients, and the incidence of patients with unknown primary tumours ranges from 0.4–38% [9]–[14]. Prognostic indicators are used to select candidates for surgery and to estimate their survival [4], [5], [8]–[10], [15]–[36]. In patients who harbour spinal epidural neoplastic metastasis, surgery is indicated for the diagnosis of the histological type of the tumour, segmental vertebrae instability and neurological deficit, particularly muscular force motor deficit [1], [5], [18], [22], [23].

In 2012, the estimated incidence of new primary cancers in Brazil was 518,510 (2.6%) in a population of 193,946,886. The overall cancer survival period statistics were imprecise because of discrepancies in death notifications [37]. There are no official government statistics for the prevalence of spinal metastases and unknown primary tumours [37]. Accessible pre-operative secondary prognostic parameters tailored for developing countries is a gap in the literature and could supplement the lack of cancer outcome data, specifically for patients with spinal epidural neoplastic metastases.

In the clinical setting with patients with unknown primary tumours and indications for surgery, secondary parameters that estimate the post-operative survival period are helpful for selecting the correct surgical procedure [10], [15]. Indices, scales and algorithms constitute evidence-based tools for survival prognostication that can contribute to decisions regarding the best therapeutic options [4], [18], [24], [29]–[33], [38], [39].

The primary prognostic parameters for survival in spinal epidural neoplastic metastases are the primary tumour and the histological tumour type [8]–[10], [15], [16], [18], [22], [23], [30], [34], [36]. When these parameters are unknown, secondary parameters are used to estimate patient survival [8]–[10], [15], [16], [18], [22], [23], [30], [36], [38]. An estimated three-month post-operative survival period seems reasonable for the decision-making process regarding whether surgery should be performed [23]. The critical judgment and clinical experience of physicians are the criteria for this decision. The surgical risks and benefits are evaluated for these patients, and the aim of treatment is the best possible quality of life during the patients’ abbreviated survival periods [5], [25]–[27], [35].

Clinically, the origin sites of histologically documented carcinomas cannot be identified in approximately 3–13% of patients, even after complete neoplasm staging [10]–[13]. In autopsy studies, 15–25% of primary neoplasms remain unidentified [11]. In 13% of spinal epidural neoplastic metastases, the primary tumour is unknown [12].

The unknown primary tumour prognostication hiatus between imaging diagnosis and definitive histological type diagnosis was the rationale for this study. Walking incapacity, special care dependency and contact between the spinal epidural neoplastic metastases and the thecal sac within the spinal canal can be used to estimate survival and can act as secondary prognostic parameter of survival for patients with spinal epidural neoplastic metastases, regardless of whether the primary neoplasm is known. The present hypothesis is that these three accessible pre-operative prognostic factors might assist in calculating a survival estimate. With accurate post-operative survival estimations, patient care might improve, allowing patients to spend more quality time with family/friends and on end-of-life planning. The authors describe these three indicators as useful survival estimation tools. In this model, no other factors were explored regarding their predictive value.

## Methods

### Data review

This research involved a retrospective review of data collected from patients who underwent surgery for spinal epidural neoplastic metastases in the Division of Neurosurgery at the Santa Casa of São Paulo Faculty of Medical Sciences between January 1997 and February 2011. The sources of the health records were written data and a chart review limited to the prognosis, diagnosis and surgical treatment. The main author collected the data.

Patients of all ages and both genders who met the following criteria were included in the study: a known survival period after surgery, spinal epidural neoplastic metastases, a Karnofsky Performance Score (KPS) [40] equal to or greater than 30 points and neoplastic metastases with a known histological type. Patients who received surgery at other institutions, with primary spine tumours and with primary spinal cord tumours were excluded. Of the 74 (100%) patients who received surgery, 52 (70%) met the inclusion criteria, and 22 (30%) were excluded from the study. A lack of post-operative hospital discharge follow-up and loss of contact with the patients or their legal guardians resulted in the exclusion of 21 patients whose home addresses and telephone numbers were unknown. The only living patient was excluded because a known post-operative survival period was a patient inclusion criterion.

The recorded data included the following information: the dates of surgery and death; registry of the surgical regime type (urgent and elective surgeries); surgical procedure data; known and unknown histological type diagnoses in the pre-operative period; walking capacity according to the American Spinal Injury Association Impairment Scale (ASIA) [41]; clinical functional performance according to the KPS [40]; anatomical relationship between the spinal epidural neoplastic metastasis and the thecal sac according to the Weinstein-Boriani-Biagini Surgical Tumour Staging System (WBB Surgical Tumour Staging System) [42]; Revised Tokuhashi Scoring System of Metastatic Spine Tumour Prognosis [31], which was calculated in the post-operative period; histological type of the spinal epidural neoplastic metastasis according to the World Health Organization Tumour Classification System; knowledge of the primary tumour site; adjuvant chemotherapy and/or radiation therapy; hospitalisation period and length of follow-up. Pathologists from the institution’s Pathology Department reviewed the post-operative tumour histological diagnoses.

In this study, survival was defined as the period from the day of surgery to the day of death, which was confirmed by documents from the hospital registry or by the testimony of family members for all 52 (100%) patients. Ambulation was defined as the capacity for independent walking with or without an aid (cane or walker). An unknown primary neoplasm was defined as a metastasis for which the tissue of origin is unknown [11], [13]. The surgical regime was established as urgent when performed within 24 hours of hospitalisation and as elective when performed after 24 hours. Neoplastic disease staging was performed in accord with the criteria established in the literature, including patient physical exams, imaging studies, laboratory tests, tumour pathology analysis and surgical reports [8], [18], [43], [44].

### Parameter contextualisation

The aim of this study was to identify accessible prognostic parameters with statistical validity for indicating post-operative survival, other than the primary tumour and the neoplasm histological type. Discrepancies in the collected data concerning prognostic parameters, such as tumour sensitivity to adjuvant chemotherapy and/or radiation therapy, visceral metastases and skeletal metastases, were incomplete in the pre-operative urgent surgery regime and inaccessible for survival prognostication; thus, these factors were not analysed statistically. Walking incapacity, special care dependency of the patient and contact between the spinal epidural neoplasms were accessible and hypothesised to be statistically significant as prognostic parameters to estimate post-operative survival in the pre-operative period. In addition to the hypothesised statistical validity, these three prognostic parameters and measurement methods were selected because they are validated measurement and classification methods in Brazilian Portuguese. They are in the public domain, familiar to medical residents and the medical staff and reproducible. In addition, they possess internal institutional validity. Our institution is a philanthropic, private, high-volume quaternary care centre that includes a teaching hospital. It was founded in 1883, and patients are referred from throughout the country and from other countries in South America. The Brazilian Basic Legislation of the Unified Health System (*Sistema Único de Saúde*) treats all patients free of charge, and hospital reimbursement is the responsibility of the federal government. All patients in this study received treatment at no charge.

The classification item *palsy* on the Revised Tokuhashi Scoring System of Metastatic Spine Tumor Prognosis [31] was modified. The ASIA Scale [41] was substituted for the Frankel Score [45]. Prof. Dr. Yasuaki Tokuhashi authorised this modification (Tokuhashi Y., personal communication, 2012). Walking incapacity and limb muscle strength were measured during the neurological exam and were expressed on the ASIA Scale [41] as A and B, with walking capacity expressed as ASIA [41] C, D and E. Pre-operative Tokuhashi Index [31] calculations were not feasible because information on the primary and secondary tumours was insufficient for 36 (69.23%) patients in this study.

The KPS [40] was used to indicate the functional performance of the patients and to infer the clinical compromise secondary to the neoplastic process. The KPS [40] allows for the classification and stratification of patients with complex clinical conditions [46].

The WBB Surgical Tumour Staging System [42] is designed for vertebral primary tumour staging and surgical strategic planning. The system is reproducible for research studies and was applied to classify the anatomical relationship between the primary or secondary neoplasm and the tissues, organs and spinal canal [42]. Magnetic resonance and computerised tomography spinal imaging situated the neoplastic metastases in the vertebrae and indicated their relationship to the thecal sac. Control post-operative spinal imaging was performed during the hospitalisation period. Vertebral segment instability was inferred in the presence of spinal epidural neoplastic metastases secondary to the vertebral segment lytic destructive process of the neoplasm. Although a specific evaluation of spinal segment instability was not within the scope of this study, we agreed with the instability criteria of the Spine Instability Neoplastic Score [19] for neoplastic metastases of the spine.

### Parameters of prognosis

Survival was individually compared with each of the following prognostic parameters: the primary tumour and its histological type (primary parameter), walking incapacity, clinical functional performance and contact between the spinal epidural neoplastic metastasis and the thecal sac (secondary parameters). Additionally, survival was compared with the combination of the three mentioned secondary parameters. The six primary tumour sites that affected only one patient each were collected in a group labelled *other*, which was included in the statistical analysis (Kaplan-Meier Method) with the seven other primary tumour sites.

### Surgical methods and indications

The authors and neurosurgeons of the Division of Neurosurgery at the Santa Casa of São Paulo Faculty of Medical Sciences performed 52 surgeries in 52 patients. Five neurosurgeons assisted by neurosurgical residents used standardised surgical methods in the patients. Spinal canal neural tissue decompression was accomplished using a dorsal or ventral spinal surgical approach. In all of the patients, spinal column stabilisation was accomplished with the use of autologous bone and/or supplementary surgical instrumentation.

The three secondary prognostic parameters offered surgical indications for spinal epidural neoplastic metastases. When the primary tumour and/or other metastatic tumours were known in the pre-operative period, the Tokuhashi Index Score [31] was calculated by summing the four following criteria:

1. The clinical state measured by the KPS [40] (equal to or greater than 30 points);
2. The neurological state measured by a fixed or progressive neurological deficit, classified as grades A, B, C or D on the ASIA Impairment Scale [41];
3. The presence of spinal epidural neoplastic metastases within the spinal canal causing nervous tissue compression, as classified by the WBB Surgical Tumour Staging System [42];
4. The survival prognosis estimated by the Tokuhashi Index [31] (equal to or greater than three points).

### Ethical considerations

The research project was approved on August 27, 2010, by the Ethics Committee on Human Research in the Santa Casa of São Paulo Faculty of Medical Sciences (protocol number 281/10). Informed oral consent was obtained from the patients’ legal guardians and conformed to the standards of the Declaration of Helsinki. The 52 (100%) research subjects included had died before the study cut-off date of February 28, 2011; thus, written consent was not obtained. The Ethics Committee on Human Research in the Santa Casa of São Paulo Faculty of Medical Sciences approved the use of oral consent.

### Statistical analysis

The Kaplan-Meier method was used to estimate the post-operative survival in the bivariate analyses of the three prognostic parameters and the histological types. The Log-Rank test was used for statistical inference. The level of statistical significance was 5% for the chi-squared distribution tests (one-tailed hypothesis). The statistical software used was the IBM *Statistical Package for the Social Sciences* (SPSS, Inc., Chicago, IL, USA), version 13.0.

## Results

### Patient demographic data

A total of 52 research subjects underwent 52 operations. There were no reoperations. The average age of the 52 patients was 53.92 years, with a range of 6–83 years (std. deviation 19.09). There were 32 (61.53%) male and 20 (38.46%) female patients. The time from the initial symptoms to the main neurological symptoms at hospital admission was 103 days on average and varied from 1 to 730 days (std. deviation 145.66). Back pain and/or pain in the limbs were the initial spinal neoplasm symptoms in 27 (51.9%) of the patients, and motor impairment was the main symptom prior to surgery in 51 (98.07%) of the patients. Hospitalisation lasted for 30.29 days on average (std. deviation 23.00). The average follow-up period after surgery was 179 days (minimum 1, maximum 993 days).

### Surgical data

The surgical procedures were performed in an urgent or elective regime for 31 (59.61%) and 21 (40.39%) patients, respectively. A stand-alone dorsal-lateral spinal column surgical access was performed in 48 (92.30%) patients, a ventral surgical access in 2 (3.85%) patients, and fluoroscopy-guided percutaneous biopsy in another 2 (3.85%) patients. Thoracic segmental tumour involvement was present in 42 (80.76%) of the 52 patients who underwent surgery. The anatomical topographic spinal neoplastic metastasis distribution was as follows: thoracic, 33 (63.46%); lumbar, 6 (11.53%); cervical/thoracic, 6 (11.53%); cervical, 3 (5.76%); thoracic/lumbar, 2 (3.84%); thoracic/lumbar/sacral, 1 (1.92%); and sacral, 1 (1.92%).

### Survival data

The average survival period was 179.38 days (95% CI 107.49–251.27), and the median survival period was 70 days (95% CI 49.97–90.02) after surgery. Tokuhashi’s [31] criterion of predicted prognosis classified post-operative survival into the following three time periods: mortality within 6 months after surgery in 38 (73.07%) patients, with 16 (30.76%) deaths occurring within the first post-operative month; between six and eleven months in 3 (5.76%) patients; and twelve months or longer after surgery in 11 (21.15%) patients. The median of the Tokuhashi Scoring System [31] was 7 points, ranging from 2–14 points (95% CI 6.46–7.66), which indicated that the expected post-operative survival after palliative surgery was less than 6 months. The post-operative median survival periods were 97 days (95% CI 69.56–124.44) for the 16 (30.77%) patients who received adjuvant chemotherapy and/or radiation therapy and 45 days (95% CI 0–92.04) for the 36 (69.23%) that did not. The adjuvant chemotherapy and/or radiation therapy cycles were incomplete for 7 (43.75%) patients and complete for 9 (56.25%) patients in this 16-patient group. The post-operative survival of the 7 patients with incomplete adjuvant therapy was less than 90 days. Because of the discrepancies of these results, the adjuvant therapy data were not statistically analysed as prognostic parameters with regard to the survival period.

### Parameters for prognosis

#### Histological data

The primary neoplasm site and histological type were unknown in 36 (69.23%) patients in the pre-operative period. The following primary tumour types were identified after surgery: myeloma (8 patients), lung carcinoma (6), sarcoma (5), prostate carcinoma (5), lymphoma (4), undifferentiated carcinoma (4), thyroid carcinoma (1), pineal germinoma (1), gastric adenoma (1) and kidney blastoma (1). The tumour prevalence is listed in Table 1.

**Table 1:** Primary sites, histological types, post-operative survival, prevalence and reliability estimates of neoplastic metastases in 52 patients.

Histological type	Median survival/days	Prevalence	CI*
Lung carcinoma	19	9 (17.30%)	14.6–23.3
Undifferentiated carcinoma	28	5 (9.61%)	6.5–49.4
Lymphoma	35	8 (15.38%)	0–26.4
Breast carcinoma	43	4 (7.69%)	0–412.4
Prostate carcinoma	45	6 (11.53%)	0–102.6
Sarcoma	72	5 (9.61%)	67.7–76.2
Myeloma	100	9 (17.30%)	41.5–158.4
Other**	502	6 (11.53%)	0–1, 004.9

*Confidence interval.

**Other: thyroid carcinoma, 506 days; pineal gland germinoma, 606 days; nephroblastoma, 877 days; suprarenal gland carcinoma, 502 days; kidney carcinoma, 87 days; and gastric adenoma, 72 days.

For the overall comparisons, the Log-Rank was used to test the equality of the survival distributions for the tumour types, with a p-value of 0.023.

#### Walking incapacity

Walking incapacity was present in 45 (86.53%) patients in the pre-operative period and in 47 (90.38%) patients after surgery. The median survival periods (measured in days) for the patients who were able to walk (ASIA [41] C, D and E) or unable to walk (ASIA [41] A and B) in the pre-operative period were 502 days (95% CI 0–1579.82) and 59 days (95% CI 12.99–105), respectively (p-value = 0.107).

#### Functional performance

The functional performance evaluation (measured with the KPS [40]) did not change between the pre-operative and post-operative periods. The scores were in the 10- to 40-point range in 47 (90.38%) patients. The scores of the other 5 (9.61%) patients varied between 50 and 70 points, and none of the patients had scores in the 80- to 100-point KPS [40] range.

The median survival periods (measured in days) in the post-operative period for the patients who were partially dependent on special care (50–70 points on the KPS [40]) and patients who were completely dependent on special care (0–40 points) were 82 days (95% CI 39–124.9) and 70 days (95% CI 33.7–106.2), respectively (p-value = 0.322).

#### Contact between a neoplastic metastasis and the thecal sac

In the pre-operative period, the neoplastic metastases were within the spinal canal and in contact with the thecal sac in 49 (94.23%) patients. They were restricted to the vertebrae in 3 (5.76%) of the 52 patients. These metastases were classified as WBB Surgical Tumour Staging System [42] anatomic sectors D and C, respectively. The control post-operative spinal imaging confirmed that there was no contact between the spinal epidural neoplastic metastases and the thecal sac.

The median post-operative survival periods (measured in days) for the patients harbouring vertebral metastases, without or with thecal sac contact, as measured with the WBB Surgical Tumour Staging System [42], were 72 days for WBB [42] sector C (95% CI 68.7–75.2) and 67 days for WBB [42] sector D (95% CI 24.4–109.5); (p-value = 0.643), respectively.

#### Combined secondary prognostic parameters

**Figure 1:**
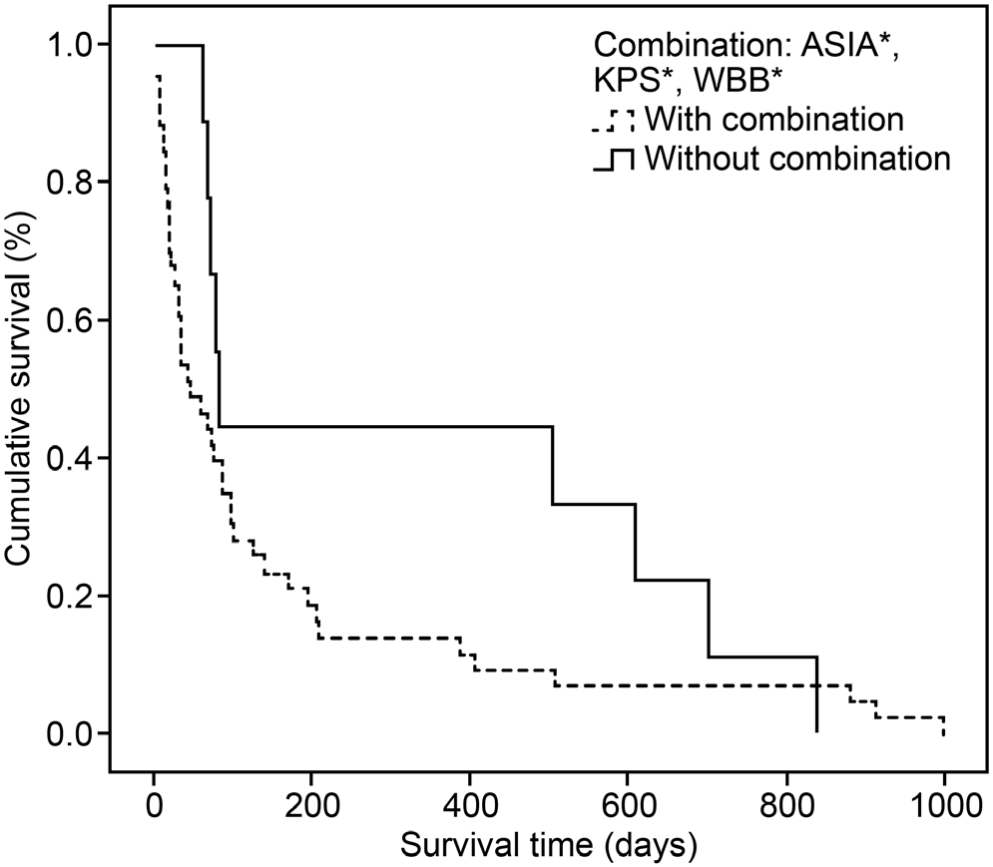
Kaplan-Meier survival curve demonstrating the survival profiles (multiple comparisons procedure). ASIA Scale [41]*: American Spinal Injury Association Impairment Scale; KPS [40]*: Karnofsky Performance Score; WBB Classification [42]*: Weinstein-Boriani-Biagini Surgical Tumour Staging System.

The patients who harboured the three secondary prognostic parameters (in combination) were compared with the patients who did not (no combination) to determine the post-operative survival of these two subgroups. In the 43 (82.69%) patients with walking incapacity, special care dependency and contact between the neoplastic metastasis and thecal sac, the median survival time was 45 days (95% CI 9.66–80.33). In the other subgroup, 9 patients (17.31%) had at least one, but not all three, of the secondary parameters of prognosis, resulting in a median survival time of 82 days (95% CI 76.15–87.84; p*-*value = 0.175) (Fig. 1).

## Discussion

Knowledge of the cancer type, tumour sensitivity to adjuvant chemotherapy and/or radiation therapy, visceral metastases and skeletal metastases are the main prognostic parameters for survival in patients undergoing a neoplastic process [4], [5], [8]–[10], [15]–[36], [38]. The rationale for this study was to identify accessible secondary prognostic parameters for use when the primary tumour is unknown. During the time interval between a spinal epidural neoplastic metastasis diagnosis by imaging and a definitive histological type diagnosis, an estimate of the period of survival allows physicians to select the best treatment, whether it is surgical or another treatment. This study differs from the published literature because of the three selected secondary parameters of prognosis and the proposal to estimate post-operative survival in the pre-operative period when the primary tumour is unknown. This study is confirmatory.

### Demographic characteristics

Compared with the populations in other studies, our patients were younger, with an average age of 54 years. The predominance of males in the sample was similar to that in other studies [13], [15], [20], [21], [28], [29], [39]. The average 3-month interval of time between the initial neurological symptoms and hospital admission was greater than the time interval in other studies [17]. The predominance of axial and/or appendicular pain as the initial symptom and motor impairment as the primary symptom agreed with the literature [8], [13], [17], [20], [29]. The average 30-day hospitalisation period was similar to the 27-day average reported by Putz et al. [29]; it was longer than the 6-day period reported by Arrigo et al. [15].

### Survival

In this series, the patients had a shorter average survival compared with that in other studies. Within the first 6 months after surgery, 38 (73.07%) of the treated patients had died. Because of delays in patient referral, the neoplastic process was frequently advanced at the time of hospitalisation, which might explain the abbreviated post-operative survival period. The following authors reported varying results for the average survival time: Yang et al. [36], 6 to 9 months; Aizenberg et al. [9], 8 months; Lau et al. [23], 10 months; Arrigo et al. [15], 8 months; Gasbarrini et al. [39], 41 months; and Holman et al. [21], 15 months.

The average post-operative Tokuhashi Index Score [31] utilised for prognostication was 7 points, in accord with the expected post-operative survival of up to 6 months. The Tokuhashi Index Score Point Interpretation [31] ranged from 1–8 points for this group of 41 (78.84%) patients. The Tokuhashi Index Scoring [31] was low and compatible with the advanced neoplastic process in most of the 52 studied patients. These findings are in accord with the literature [29], [32], [34]. The Tokuhashi Index Scores [31] we obtained confirmed their predictive capacity in this study when compared with the overall post-operative survival of our patients, which was 6 months. We agree with Oliveira et al. [3] that the Tokuhashi Index Score [31] for pre-operative prognostication was not useful in guiding the treatment type because in the majority of patients with vertebral metastases, their pre-operative prognostication variables were incomplete.

All 52 (100%) of the research subjects died. Death occurred in 41 (78.84%) of the 52 patients between 0 and 12 months, which might explain the shorter period of post-operative follow-up compared with those in other series. Follow-up was conducted for more than 11 months in 11 (21.15%) of the patients. A post-operative patient follow-up period of approximately 10 months has been reported in the literature [17]. Lau et al. [23] reported the results of a 2-year retrospective post-operative follow-up of 99 patients from 2005 to 2011.

Sixteen (30.76%) patients received adjuvant chemotherapy and/or radiation therapy in 9 complete and 7 incomplete cycles. This small number of patients submitted to adjuvant therapy likely resulted because 38 (73.07%) of the patients had advanced neoplastic processes and survived less than 6 months after surgery. The median post-operative survival period was longer for the 9 patients who completed the adjuvant chemotherapy and/or radiation therapy cycles than those who completed the surgical series, 97 days (95% CI 69.56–124.44) and 70 days (95% CI 49.97–90.02), respectively. This small difference suggests a tendency for a longer post-operative survival period when surgery and adjuvant therapy are associated in the treatment plan. The literature supports this point of view [4], [18], [22], [23], [37], [39]. Hirabayashi et al. [20] stated that 75 (70%) of the 107 patients they studied from 1985 to 2001 were subjected to adjuvant therapy and underwent surgery for spinal epidural neoplastic metastases, and these patients had a 10-month survival period.

### Histological data

Cancer type was the strongest survival indicator in this study (p-value = 0.023). The tumours were analysed as a group for overall survival and compared with the secondary prognostic parameters. As expected, the primary tumour site and histological type of the neoplasm were significant as primary prognostic parameters because of the biological characteristics of each neoplasm, which were specific to each patient.

The primary tumour was unknown before surgery in 36 (69.23%) of the patients because of incomplete neoplasm staging prior to elective surgery or urgent surgery. A definitive histological type diagnosis was obtained in the post-operative period in all 52 (100%) patients using a multidisciplinary neoplasm staging work-up.

When the median survival period after surgery was compared between the shortest and the longest life spans, the patients with lung carcinomas survived for 19 days on average (95% CI 14.6–23.3), whereas those with myelomas survived for 100 days (95% CI 41.5–158.4). Lung carcinoma exhibited the most aggressive biological behaviour in our series. These findings are in accord with the existing literature [20], [34].

### Waking incapacity

Walking incapacity was the strongest secondary parameter of prognosis in this series (p-value = 0.107). The capacity to walk implies a capacity for self-care. The authors observed that the 47 (90.38%) post-operative patients who could not walk were dependent on institutional care for personal hygiene, feeding and locomotion and had aggravated neoplastic disease. These patients fared worse than those who could walk.

Walking capacity has been used as a secondary parameter of prognosis in the post-operative period in the literature [8], [20], [30], [31], [35], and in this study, pre-operative walking incapacity was used. The ASIA Scale [41] was used instead of the Frankel Index [45] to ensure accuracy and precision in the results, which were reproduced throughout the study. The ASIA Scale [41] measures the motor and sensitive functions (accuracy) and the motor and sensitive S4 and S5 sacral segment functions (precision) [41]. Putz et al. [29] measured motor deficiencies using a modified ASIA Scale [41]. Other authors have used the Frankel Index [9], [12], [15], [20]–[22], [28], [31], [45] or have omitted motor deficiency measurements [10], [23], [33], [38].

### Functional performance

Patient special care dependency (KPS [40]: 0–40 points) was the second most relevant of the three secondary prognostic parameters (p-value = 0.322). This study indicated that the lower the point sum was, the higher the grade of clinical need caused by the neoplastic process was. In this series, all of the patients were hospitalised, disabled and reliant on active vital support for life. As such, walking incapacity might have contributed to their limited survival. None of the 47 (90.38%) patients improved in their functional status in the post-operative period.

The KPS [40] is used as a prognostic factor in patient assessment to measure and compare the functional statuses of individual patients [46]. In the literature, the KPS [40] is inserted in the Revised Tokuhashi Scoring System of Metastatic Spine Tumour Prognosis [24], [29], [31] or is used as an independent method of quantifying functional performance [12], [16], [34].

### Contact between the neoplastic metastases and the thecal sac

Contact between the neoplastic metastasis and the thecal sac was the weakest of the three secondary prognostic parameters in this study (p-value = 0.643). This parameter has not been previously reported in the literature as a prognostic parameter. The vertebral neoplastic commitment is itself a prognostic factor, and the neoplastic thecal sac contact might not be as relevant as hypothesised in this study.

### Primary versus secondary prognostic parameters

When the primary parameter of prognosis is unknown, we propose the use of secondary parameters of prognosis. The secondary parameters of prognosis, in decreasing order of statistical relevance for post-operative survival, were as follows: impaired walking (ASIA [41] A and B), 59 days (95% CI 12.99–105); special care dependency (KPS [40] 0–40 points), 70 days (95% CI 33.7–106.2); and contact between the spinal epidural neoplastic metastasis and thecal sac within the spinal canal (WBB Surgical Tumour Staging System [42] sector D), 67 days (95% CI 24.4–109.5).

When these three parameters were combined, the mean post-operative survival was 45 days (95% CI 9.66–80.33); when at least one parameter was present, the survival was 82 days (95% CI 76.15–87.84). The reason for this difference of 37 days between the two subgroups was not identified; however, the biological behaviour of the primary tumour is presumed to be the most important prognostic factor. The combined secondary parameters of prognosis (p-value = 0.175) indicated the probability that the observed statistics occurred only by chance in at least 17% of the cases, in contrast to 2% for the primary parameter of prognosis. These secondary prognostic parameters reflect a trend towards predicting survival in this model; however, these parameters might only reach significance upon testing a larger sample size, which should be addressed in future studies.

The median post-operative survival for the 16 (30.77%) patients with known pre-operative primary tumours was 59 days (95% CI 11.96–106.04), and it was 72 days (95% CI 51.45–92.54) for the 36 (69.23%) patients with unknown tumours. The reason for this difference was not identified, and the 52 research subjects were statically analysed as a whole, thus validating the use of the aforementioned 3 prognostic parameters for known and unknown primary tumours (p-value of 0.023).

There is no sense of prejudice or subjectivity for deviation from the truth, and the prognostication results are incidental because of the small sample groups; however, these parameters are considered a useful prognostication tool.

### Surgical data and indications

A 2- to 3-month post-operative survival period justified surgical treatment in our series of 52 patients, although this time period is clearly an abbreviated post-operative period of survival. Surgical treatment was tailored to the dying patient and aimed to improve the quality of their last days. Post-operative nursing care for a surgically stabilised spinal segment facilitated patient bed mobilisation, personal hygiene and transportation between hospital facilities and home.

These authors agree with the following criteria for surgical indications developed by Lau et al. [23]: life expectancy of at least 3 months when the primary tumour is known, imaging evidence of spinal neoplastic metastasis, neurological deficit and intractable pain and/or concern for instability.

Installed paraplegia within 24 hours and rapid progressive paraparesis were the indications for urgent surgery performed within 24 hours of hospitalisation in 31 (59.61%) of our patients. None of the patients regained walking capacity within the first post-operative week. These patients might have experienced improvements in walking impairment if their survival had been longer than the median 70 days; for this reason, surgery was performed. Other indications for surgery in this series, in accord with the literature, were as follows: spinal segmental instability, intractable pain and diagnosis of the histological type of neoplastic metastasis [1], [4], [8], [18], [22].

The limitations of this study, including its retrospective nature, small sample size and long time interval (14 years), are outweighed by its internal institutional validity and the beneficial use of the 3 proposed prognostic parameters in a series with 69.23% unknown primary tumour pre-operative patients. The results are not statistically significant, and the three identified prognostic parameters are known in the literature [8], [18], [24], [31], [32], [35], [42]. Treatment was not withheld from the research subjects that had the surgical indications listed in the methods section. Comparisons between operated versus non-operated patients were not the focus of this study. As the study continues, the authors’ aim is to compare the two groups, and the authors are aware that predictive factors are statistically worthy when they are compared to a control group. Larger prospective studies are needed to address these limitations.

## Conclusions

The results of this study allow an accurate estimate of survival for patients with spinal epidural neoplastic metastases with unknown primary tumours in a pre-operative urgent regime. The intended implications of this study were to predict the survival and criteria for surgical indications. In addition to knowledge of the cancer type, the following three identified prognostic factors were useful in predicting survival: walking incapacity, special care dependency and contact between the neoplastic metastasis and the thecal sac. These factors could facilitate the determination of the ultimate survival of this patient population and the identification those who would benefit from surgery versus palliation. For a patient with an advanced neoplastic process and an unknown primary tumour, forecasting an abbreviated 2- to 3-month survival estimate indicates surgery and could increase the quality time that patients have to spend with family and friends and on end-of-life planning. With respect to the surgeon facing this critical clinical situation, he or she might be more comfortable performing a suitable palliative surgery that aims to stabilise the committed spinal segment and obtain a histological tumour type diagnosis with the aforementioned statistical indications. This study could be included in future cohort studies for meta-analysis. Multicentre trials are necessary to determine the future guidelines and standards for survival prognostication.
